# Success Rates of Zygomatic Implants for the Rehabilitation of Severely Atrophic Maxilla: A Systematic Review

**DOI:** 10.3390/dj10080151

**Published:** 2022-08-12

**Authors:** Aleix Solà Pérez, David Pastorino, Carlos Aparicio, Marta Pegueroles Neyra, Rabia Sannam Khan, Simon Wright, Cemal Ucer

**Affiliations:** 1 Zygoma ZAGA Centers, Carrer Castellá 33, 08018 Barcelona, Spain; 2 Clínica Hepler Bone, Román Macaya 22, 08018 Barcelona, Spain; 3 Biomaterials, Biomechanics and Tissue Engineering Group, Department of Materials Science and Engineering, Universitat Politècnica de Catalunya (UPC), EEBE, 08019 Barcelona, Spain; 4 ICE Postgraduate Dental Institute and Hospital, Salford M50 3XZ, UK; 5 School of Health and Society, University of Salford, Salford M5 4WT, UK

**Keywords:** dental implants, zygomatic implants, atrophic maxilla, cumulative success rate

## Abstract

Zygomatic implants are a treatment solution for patients with severe maxillary atrophy. This treatment option allows delivering immediate fixed teeth within 24 h. Numerous peer-reviewed publications have reported different success rates, resulting in a disagreement on the topic. Therefore, the overall efficacy and predictability of this rehabilitation is still a matter of discussion. With this study, we aimed to identify the published literature on the use of zygomatic implants for the reconstruction of the severely atrophic maxilla and report the cumulative success rate (CSR) as a function of follow-up time. A systematic review of the literature on zygomatic implant for the treatment of severe maxillary atrophy was performed and 196 publications were included in the study. The cumulative success rate of zygomatic implants for the treatment of severe maxillary atrophy was 98.5% at less than 1 year, 97.5% between 1 and 3 years, 96.8% between 3 and 5 years and 96.1% after more than 5 years. The most commonly reported complications were soft tissue dehiscence, rhinosinusitis and prosthetic failures. The treatment of severe lack of bone in the upper maxilla with zygomatic implants is a safe procedure, reaching a cumulative success rate of 96.1% after more than 5 years.

## 1. Introduction

Zygomatic implants have stood out in the field of dental implantology as an immediate solution to severe maxillary atrophy. In regard to the local anatomy of the maxilla and dimensions of sinus cavity, several approaches have been recommended for the rehabilitation of the maxilla with tilted implants, combined with bilateral/single zygomatic implants. After local infection, severe bone resorption and oncological resective surgery, zygomatic implants propose a valuable treatment. Zygomatic implant (ZI) therapy is an elective treatment modality for the rehabilitation of partially and fully edentulous patients, pioneered by Branemark et al. in 1998 [1]. It was proposed that zygomatic implants are an evidence-based surgical and prosthetic solution for both two-stage and immediate loading protocols, using either intra-sinus or extra-sinus approaches [2]. Their use is widespread, ranging from trauma and oral cancer rehabilitation cases to failed conventional implant cases, an alternative to augmentation in the atrophic maxilla and failed augmentation. Zygomatic implants can be placed in different ways: bilateral two implants (quad), bilateral one implant, unilateral one or two implants and in conjunction with conventional dental implants [2].

The need for zygomatic implants arises from the impossibility of placing regular implants due to the lack of adequate residual bone. Indeed, zygomatic implants offer a definitive solution to lack of bone by allowing the placement of implants into zygomatic bone to anchor full arch prostheses. The treatment of severe maxillary atrophy with zygomatic implants is a solution that enables the delivery of an immediate provisional prosthesis within 24 h of surgery [3,4]. This is particularly relevant when compared with alternative options such as bone grafting, where the time to the prosthesis is substantially longer [4]. The benefits of an immediate solution are critical for patients’ quality of life.

The zygomatic implant (ZI) positioning avoids the maxillary sinus lifts of onlay block grafting, which are indicated when reconstructing the severely atrophic maxilla with autologous grafts harvested from the iliac crest, calvaria or intra-orally from the mandible. Zygomatic implant surgery generally results in less severe symptoms or morbidity when compared with grafting options, which result in higher surgical morbidity, with reduced predictability, higher treatment costs and prolonged overall duration of treatment. The zygomatic bone articulates with sphenoid bone, maxilla, frontal bone and temporal bone to form the lateral wall of the floor of the orbit and contributes to the formation of the temporal and infratemporal fossa bilaterally, characterized by trabecular and cortical components that are ideally positioned to provide robust anchorage points for zygomatic implants [4].

Although the ZI technique has been used over the past 30 years, with reports of good clinical success rates [5], currently, there is a lack of universally recognized success criteria for zygomatic implant rehabilitation, which strongly hinders the long-term follow-up of this important treatment modality. Although two success criteria have been proposed, namely, the Zygomatic Success Code (Aparicio et al., 2000) [6] and the ORIS criteria of success [7], their use in systematically reporting results is limited. The ORIS criteria is a refined version of the Zygomatic Success Code, including four dimensions in the definition of success:

- Offset measurement as an evaluation of prosthetic positioning.
- Rhino-sinus status report based on a comparison of presurgical and postsurgical cone-beam computed tomography and a clinical questionnaire.
- Infection permanence as an evaluation of soft tissue status.
- Stability report, accepting as success some mobility until dis-osseointegration signs appear.

To evaluate the level of clinical validation and expected outcomes of zygomatic implant rehabilitation over time, we report the success rate over time, as defined by the authors of each reviewed study. The success is evaluated at one or several follow-up times points according to each author’s documented criteria and reported in this work as the Cumulative Success Rate (CSR) at the follow-up time.

Zygomatic implant indications include severe maxillary atrophy as well as oncology reconstruction. The type of procedure studied may include between 1 and 4 zygomatic implants. Whenever possible, the cumulative success rate is extracted from the peer-reviewed publication, selected and aggregated. The objective of this work was to review the state of the art and report the cumulative success rate of the treatment of maxillary trophy with one or several zygomatic implants, in combination eventually with other types of implants. Due to the high variety of reporting systems, the criteria of success used is the one chosen by the authors of each publication [8,9].

The evolution and development of the treatment with zygomatic implants, as well as the cumulative success rate as a function of the follow-up period, must be studied and reviewed. At present, the way in which zygomatic implants are discussed in the literature lacks a standardized systematic review. There are many inconsistencies at a basic level, from the definition of terms used to categorize the data success, survival and failure. They are described and evaluated in the same way that conventional dental implants are, when they are inherently different in design, biomechanics and the techniques of placement. The complications and duration of the potential complications need to be placed into categories which will separate survival and failure.

Zygomatic and maxilla measurement is required for the pre-surgical planning of implant fixation, and it requires 5.75 mm of zygoma thickness for 3.75 mm of implant to be placed with the angulation of 43.8° or even less. There is an increased risk of perforation into the infratemporal fossa, as well as the angulation of 50.6° might increase the risk of perforation on to the orbital floor, which was showed in a study conducted by Uchida et al., 2001. The maxilla atrophies have been classified by Cadwood-Howell from class I to VI as follows to understand the level of atrophy in order to plan the treatment: (I) teeth present, (II) immediate post-extraction socket, (III) edentulous ridge with adequate height and width of bone, (IV) knife edge ridge and adequate height but inadequate in width, (V) flat bone ridge bone inadequate in width and height and (VI) depressed from ridge basal bone resorption. The zygomatic implant insertion path is from the alveolar ridge starting from second premolar or first molar region, passing through the maxillary sinus or the wall into zygomatic bone. The implant body will be inserted into the thicker and wider cancellous bone of zygoma.

## 2. Materials and Methods

### 2.1. Selection of Articles

This systematic review focuses on patients who needed oral rehabilitation with zygomatic implants in severe maxillary atrophies. The intervention under consideration was zygomatic implant placement for immediate maxillary rehabilitation.

The search protocol used the electronic databases PubMed, Google Scholar and Scopus with a time limit from 2000 to 2022. The latest database complete search was performed in March 2022. The search strategy utilized a combination of medical search terms (MeSH) and keywords including (survival rate) AND (zygomaticus implants) AND (zygomatic dental implants)) OR (maxillary atrophy) AND (Atrophy/pathology) OR (Dental Prosthesis, Implant-Supported*) AND (Zygoma/surgery*) AND (Aged)) AND (Adult) AND (Maxilla/surgery) OR (Risk Factors) AND (Jaw, Edentulous/surgery*) OR (Dental Implantation, Endosseous/methods*).

The study’s search criteria were defined according to the Preferred Reporting Items for Systematic Review and Meta-Analysis (PRISMA) guidelines. Only peer-reviewed articles written in English were selected. Three reviewers screened the articles independently.

To identify studies, abstracts and titles were screened followed by full-text articles, which were specifically limited to randomized clinical trials, systematic reviews and metanalyses, and prospective and retrospective studies following the guidelines of CSR reporting with zygomatic rehabilitation.

### 2.2. Inclusion and Exclusion Criteria

The inclusion criteria were limited to publications covering full or partial fixed maxillary rehabilitation, severe maxillary atrophy and pathological and ongoing illness patients, including zygomatic implants using delayed or immediate-loading protocols. We considered studies if they matched the following description, as proposed by the Cochrane Collaboration Handbook (Chapter 1.2.2) [9]: “It uses explicit, systematic methods that are selected with a view to minimizing bias, thus providing more reliable findings from which conclusions can be drawn and decisions made.” The outcome variables were the following: (A) cumulative success rate (CSR), implant survival/failure; (B) complications (surgical/prosthetic). The exclusion criteria included letters to editors, laboratory modeling or in vitro studies.

To identify studies, abstracts and titles were screened, followed by full-text articles, which were limited to randomized clinical trials, systematic reviews, meta-analyses, prospective and retrospective studies and case reports.

### 2.3. Assessment and Classification of Studies

The selected studies were classified as clinical publications, reviews and meta-analyses and others such as future developments. The clinical publications were further separated according to the follow-up period stated, separating in less than one year, from 1 to 3 years, from 3 to 5 years and more than five years.

The clinical studies were then assessed, and critical information was extracted: the number of treated patients, the number of zygomatic implants and the cumulative success rate.

The articles identified as reviews were analyzed, and information on follow-up periods, number of patients and cumulative success rate was extracted. Studies that do not contain this information were not included in this work.

### 2.4. Reporting the Success of Zygomatic Implant Rehabilitations

The criteria of success for zygomatic implant rehabilitations vary among publications. Therefore, the cumulative success rate (CSR) was extracted from each study, followed by each author’s measures of success. The variations in success criteria among authors are hypothesized to be compensated by the high number of publications included in this work.

## 3. Results

### 3.1. Search Results and Classification

**Figure 1:**
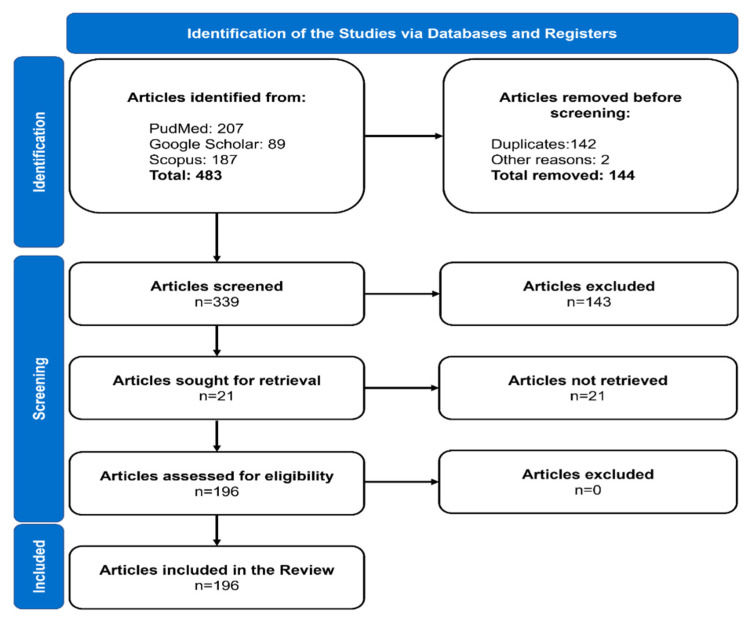
Summary of selection of studies process by following the PRISMA guidelines.

The articles used for this review after the search and the application of inclusion and exclusion criteria are represented in Figure 1 following the PRIMSA guidelines.

**Figure 2:**
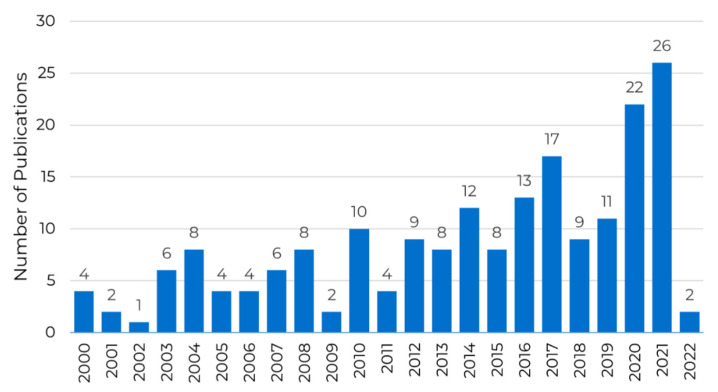
Number of publications per year from 2000 to now, showing the publication trend over the years.

In total, 339 studies were identified, and 143 were excluded due to being in vitro studies or laboratory models and not meeting the inclusion criteria. As a result, 196 articles were used for this review, and the evolution of the volume of the selected publications over time is shown in Figure 2.

The number of scientific articles related to zygomatic implants for treating severe maxillary atrophy shows an increasing trend over the years. There were no more than ten articles per year until 2012, showing a strong increase in 2020 and 2022. The recent increase in publications reflects the growing clinical interest in offering this treatment option to patients suffering from severe maxillary atrophy.

Dr. Aparicio appeared to be the most active author, with 17 articles from 2000, followed by Dr. Davó, with 15 publications. Furthermore, Dr. Peñarrocha-Diago and Prof. Malevez have also been very active in research in this field, with 11 and 10 articles published, respectively. Moreover, Dr. Wu, with nine publications; Dr. Wang and Dr. Nobre, with seven publications each; and Dr. Maló and Dr. Bedrossian, with six publications, have shown themselves as the most active authors in the field of zygomatic implants as a solution to severe maxillary atrophy.

**Figure 3:**
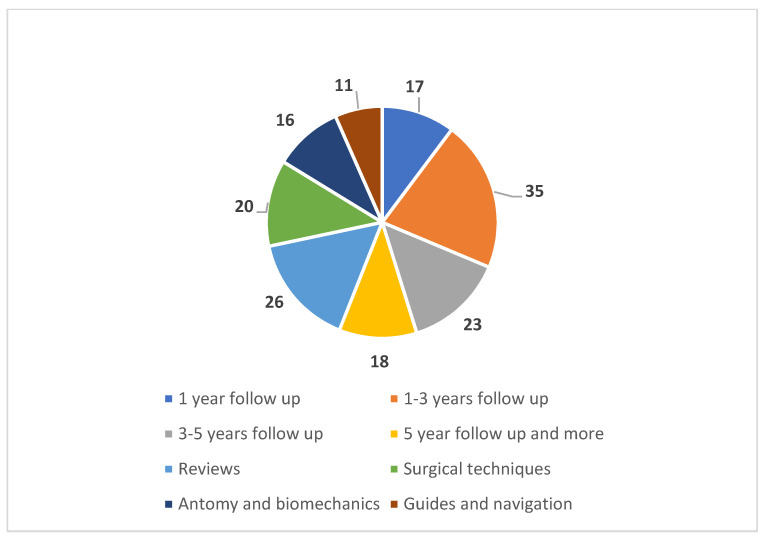
Classification of the 196 selected articles compiled according to their theme, separating them into three large sections: clinical cases, reviews and others.

The clinical publications were further split according to the follow-up period of the clinical cases (Figure 3). The other publications were grouped according to the topic covered. The publications are classified as 93 clinical publications, 26 reviews and 77 others.

### 3.2. General Properties of the Studies Included

The studies were published from 2000 to 2022 on the qualitative analysis of the effectiveness and survival rate of zygomatic implants. The survival rate was found to be higher; however, heterogeneity was observed in terms of study designs, study population, the protocols for surgery and implant geometries. The habitual factors of patients, age, gender, survival criteria, follow-up time, implant loading timing, loading angles and prosthesis are involved in the survival success rate of zygomatic implants, which varied among the selected studies (Table 1).

**Table 1:** General characteristics of the studies included for the *evaluation*. % of soft tissue dehiscence, rhino-sinusitis and prosthetic failures.

Authors/Year	Patient Age and Gender	Habits	Settings and Location	Reason for ZI	No. of Zygomatic Implants	Type of Prosthesis	Follow-Up	Survival Rate, Success %	Implant Failure	Complications
Hirsch et al., 2004 [9]	76	Non-smokers	Sweden	Ongoing illness	145 zygoma fixtures at 16 centers	Prosthetic bridge and zygoma fixtures	1-year follow-up	97.9% after 1 year	9.1% over the period of 80 months	Excessive bleeding, postoperative infection, pain, impaired nerve function, unilateral paresthesia, fistula formation, mucositis, abutment screw fracture, framework fracture
Aparicio et al., 2008 [10]	47 zygomatic in 25 consecutive patients, (12 females, 13 males, mean age 48 years, range 34–78 years)	13 patients were smokers and 12 were diagnosed as bruxers	Spain	Less than 4 mm of available bone height and width	47 zygomatic implants	Immediate/early loading	Follow-up controls were performed at 1, 4 and 12 months, annually from 2 to 5 years	Cumulative survival rate 100%	1 fracture only	Anterior teeth fractured in 5 patients with metal resin (*n* = 4) and metal porcelain (*n* = 1) bridges and one abutment screw fracture
Davo et al., 2008 [11]	19 men and 23 women (42 patients) mean age of 57 years (range: 34 to 79 years)	The exclusion criteria for use of ZI were acute sinusitis and heavy smoking (more than 10 cigarettes per day), bruxism, uncontrolled diabetes and metabolic diseases	Department of Implantology and Maxillofacial Surgery, Medimar International Hospital, Alicante, Spain (2 years)	37 patients were totally edentulous and 5 were partially edentulous	Fixed prosthesis screwed onto implants within 48 h of implant placement	1 year follow-up	The success criteria for the ZI were (1) implant anchorage to the zygomatic bone confirmed by cranial radiograph; (2) the implant anchoring for the functional prostheses; (3) no pain, suppuration, pain or pathology at maxillary and zygomatic level; (4) implant stability confirmed 100% success rate	100%	0%	Oroantral fistula and sinusitis
Chow et al., 2010 [12]	16 patients, 9 females and 7 males with mean age of 60	2 male smokers	China	Severely atrophic maxilla	37 zygomatic implants	6 and 12 months	90.3%	Fixed prosthesis, clinical stability and no sinus infection	9.7%	Sinus infection
Stie´venart et al., 2010 [13]	20 patients (mean age 56 years)	Non-smokers	Department of Maxillofacial Surgery and Dentistry, Erasme Hospital, Free University of Brussels, Belgium	Extremely resorbed maxilla	4 zygomatic implants	Fixed bridge	3 years	The survival rate of the implants after 3 years is 96% (77 implants of 80)	4%	Sinusitis, cheek bone hypoesthesia, soft tissue inflammation
Migliorança et al., 2011 [14]	75 patients with severely atrophic maxillae (mean age 52 years)	Non-smokers	Campinas, São Paulo, Brazil, between 2003 and 2006	Rehabilitation of the edentulous maxilla	150 zygomatic implants	Screw-retained prosthesis	3 years of follow-up	2 zygomatic implants were removed		
Malo et al., 2012 [15]	39 patients (30 women and 9 men), with a mean age of 53 years	Non-smokers	Private rehabilitation center between January 2006 and October 2009	Completely edentulous maxilla rehabilitation	92 zygomatic and 77 regular implants	Fixed prosthesis	3 years follow-up	82%	18%	5 cases of sinusitis and 1 oro-antral communication
Penarrocha et al., 2017 [16]	21 patients, 11 women and 10 men, mean age 54	3 male smokers, 1 high blood pressure female, 1 male ectodermic dysplasia	Valencia, Spain, from 2000 to 2005	Maxillary atrophy	40 zygomatic implants from 2000 to 2005	Screwed fixed prosthesis	Annual follow-up	Success criteria was if after implant placement there was no infection, pain or mobility, and is able to support the prosthesis		Sinusitis, ecchymosis, 2 implant failures
Esposito et al., 2018 [17]	35 edentulous patients	22 non-smoker and 13 smokers	Barcelona, Spain; Malpighi, Bologna and Rome, Italy	Atrophic edentulous maxilla and not having sufficient bone volumes	4 ZI in severely atrophic maxillae 2 zygomatic implants per side were placed and immediately loaded, February 2012 to September 2015	Immediate loading, screw-retained, metal-reinforced, acrylic provisional prostheses with ceramic or acrylic veneer materials	4 months after prosthetic loading	The mean number of days to have a functional prosthesis was 444.32 ± 207.86 for zygomatic patients, the difference being statistically significant (mean difference = −442.9; 95% CI)	1 patient lost 3 implants vs. 35 implants in 8 patients	Sinus epithelium perforation, peri-implantitis, infection, nasal floor, sinusitis, periorbital infection, fistula
Pellegrino et al., 2020 [18]	20 patients were recruited The mean age was 64.9 ± 11.5 in the atrophic group and 66.5 ± 13.6 in the oncologic group	Data not available	University of Bologna from October 2013 to January 2019	Need for maxillary rehabilitation Lack of bone height in the maxillary posterior region due to pneumatization of the sinus and/or resection for cancer	Severe maxillary atrophy (10 patients) and bone defects in oncologic patients (10 patients)	Screw-retained prosthesis	39.9 months	The 5-year implant survival rate for patients with maxillary atrophy and oncologic patients was 97.4% and 96.7%; the prosthetic survival rate was 100%	2 implant failures occurred in the first year	Prosthetic screw fractures, chipping and fracture of abutments
Duarte et al., 2020 [19]	12 patients between January 2017 and January 2020; 8 women and 4 men, the average age for the women being 61 ± 9 years and for the men 59 ± 14 years	Non-smokers	Portugal	Edentulous maxilla	2 zygomatic implants (S.I.N. Implant System, São Paulo, Brazil) placed bilaterally, combined with two short implants placed in the pre-maxilla (S.I.N. Implant System, São Paulo, Brazil); 24 zygomatic implants	Fixed provisional acrylic prosthesis attached 5 to 6 h after surgery	12 to 60 months	Implant survival rate 100%	0%	2 infective complications were detected in 2 patients who each lost 1 implant, severe infection of the maxillary sinus
Wang et al., 2021 [20]	15 patients (3 men, 12 women; age range 19–71 years; average age 47.2 years)	N/A	USA	Maxillary edentulous, adequate in height and inadequate in width	ZI quad approach from January 2017 to January 2020; 13 of 15 patients (86.7%) received immediate loading	Permanent prosthesis	Mean follow-up of 17.2 ± 6.2 months	13 of 15 patients (86.7%) received immediate loading	2%	Sinusitis

### 3.3. Cumulative Success Rate of Zygomatic Implants

**Figure 4:**
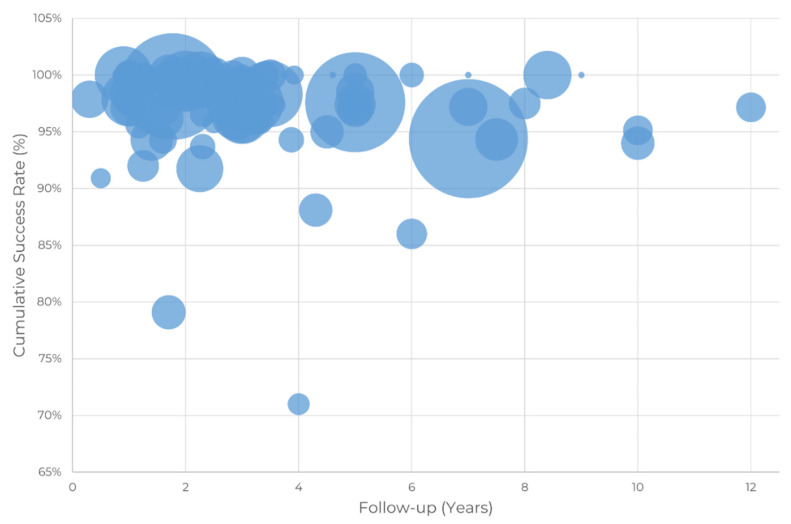
Cumulative success rate as a function of clinical follow-up. The size of the circle is proportional to the number of patients included in the study.

**Figure 5:**
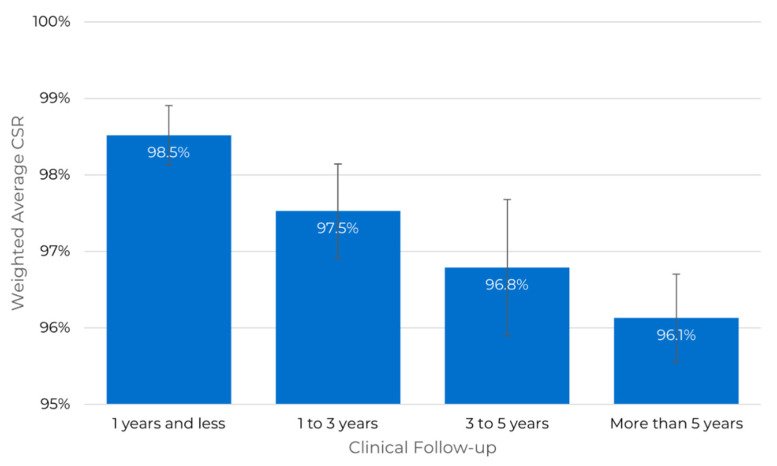
Average CSR (weighted mean using the patient count) as a function of clinical follow-up. The error bar represents the standard deviation of the weighted mean to account for each group’s different number of studies.

The CSR is an indicator of the success of zygomatic implants as a solution to severe maxillary atrophy. The 93 peer-reviewed publications identified as clinical publications were analyzed, and the CSR of the procedures reported by the author(s) as well as the number of patients included in the study were extracted and are summarized in Figure 4 and Figure 5.

Figure 4 shows that most studies include a short clinical follow-up period. Long-term studies are scarce. Moreover, we can observe a tendency that CSR decreases with clinical follow-up time in Figure 5.

The CSR up to one year of clinical follow-up for the 411 patients included in the study is 98.5%, with a standard error of the weighted mean of 0.4%. The reported success rates range between 97.2% and 100.0%, with one outlier by (Hinze et al., 2013) [21] deviating down to a 90.9% success rate. This period includes 17 publications [5,11,20,21,22,23,24,25].

The CSR between 1 and 3 years of clinical follow-up for 1229 patients included in the studies is 97.5% with a standard error of the weighted mean of 0.6%. This period included 35 publications. The CSR between 3 and 5 years of clinical follow-up for 656 patients included in the studies is 96.8% with a standard error of the weighted mean of 0.9%. This period included 23 publications. After more than five years of clinical follow-up, the CSR for 1025 patients included in the studies is 96.1%, with a standard error of the mean of 0.6%. This period included 18 publications [26,27,28,29].

Complications were frequently reported. It remains unclear whether they are the cause of the reported failures. Soft tissue complications were reported in 34.7% of cases. Rhinosinusitis represented 33.7% of the complications, while prosthetic complications accounted for 17.8%. Finally, implant stability and integration corresponded to 8.9% of complications.

Twenty-six reviews and meta-analyses were identified. Among them, 16 included CSR after a certain clinical follow-up period. Table 2 summarizes the CSR, range of clinical follow-up and the number of patients included.

**Table 2:** CSR (%), number of patients and years of follow-up for reviews.

Review Publication References	CSR (%)	Number of Patients	Clinical Follow-Up (Years)
(Gracher et al., 2021) [30]	98.2	1247	0–19
(Ramezanzade et al., 2021) [31]	95.2–100.0	-	10
(Muñoz et al., 2021) [32]	99.3	921	0.3–10
(Lan et al., 2021) [33]	96.0–100.0	166	0.5
(Lorusso et al., 2021) [34]	94.1–100	1430	0.5–8
(da Hora Sales et al., 2020) [35]	96.7	2313	5.4
(RM et al., 2019) [36]	97.8	-	1–10
(Alqutaibi and Aboalrejal 2017) [37]	95.2	2161	12
(Alejandro et al., 2016) [38]	98.6	738	0.5–5.8
(Chrcanovic, Albrektsson and Wennerberg 2016) [39]	95.2	2161	12
(F. Wang et al., 2015) [40]	96.7	49	2.5–2.8
(Goiato et al., 2014) [41]	97.9	748	3.5
(Vashisht, Bhalla and Prithviraj 2014) [42]	>90.0	418	0.5–6
(Chrcanovic and Abreu 2013) [43]	96.7	1145	12
(Candel-Martí et al., 2012) [44]	97.1	486	1–10
(Galán Gil et al., 2007) [45]	82.0–100.0	312	0.5–10

## 4. Discussion

This work focuses on reporting the CSR at different clinical follow-up periods of zygomatic implants used in the rehabilitation of patients with severe maxillary atrophy. ZI is a highly sensitive treatment and classed as a major surgery with many anatomical risks to be taken into consideration. This may well introduce some bias to the results as the surgeons carrying out this treatment modality are few. With highly skilled surgeons carrying out the treatment, it could be argued that their level of surgical skill is superior to those carrying out conventional approaches, and therefore, there is a risk of bias while extrapolating their results. Therefore, it is challenging and time-consuming to carry out investigations and research with only one surgeon operating.

The studies varied from single center to multiple centers, from one surgeon to multiple and from 1 year of follow-up to 3 years. The different survival criteria could give different survival rates in the studies; however, consensus on one survival criteria is necessary to gauge the survival of zygomatic implants. The definition of "success" is a critical hypothesis in this analysis. Indeed, while most papers consider success as the survival of the implants placed, other authors link it to prosthetic success, patient satisfaction and quality of life over time. Although the need for harmonized, clinically applicable criteria of success for zygomatic implants is clear, it is also essential to note that the definition of success for alternative treatments, such as bone block grafting, meets the same challenges. The use of the ORIS success code can represent a harmonized solution matching the patient-focused philosophy that dentistry has adopted in recent years. The scope of this work is limited to reporting success as defined by the authors of each publication.

The CSR over time tends to decrease. Significant variability is observed among publications, with CSR as low as 71% at four years (Landes 2005b) and as high as 100% at five years (Davó and Pons 2015) [46]. Thus, the CSR was studied in four different clinical follow-up periods, leading to two main conclusions: the CSR decreases with clinical follow-up time, and the CSR remains greater than 96% after five years of clinical follow-up.

**Figure 6:**
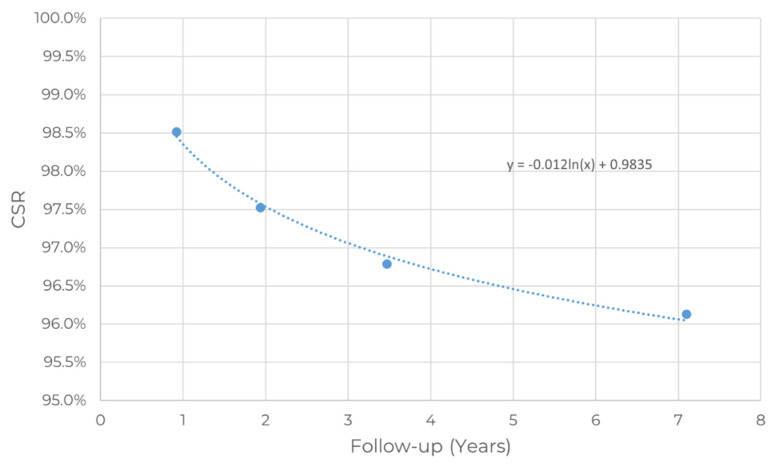
Regression of the CSR(%) as a function of the follow-up period.

A regression of the data in Figure 6 strongly suggests that a logarithmic model fits the data well, with a coefficient of determination R2 of 0.993 using the following equation:

CSR(%) = 0.9835 − 0.012.ln(Follow-up (years))

Given the logarithmic nature of the equation, it suggests failures are more prone to occur in the early years than in the long term.

Continuous scientific and clinical research on zygomatic implants supports the high CSR and its decrease with time. Complications including rhinosinusitis and soft tissue dehiscence are reported. Although several authors studied the correlation between decreasing CSR and complications, further studies, including multifactorial definitions of success, are required [47].

A thorough understanding of the anatomy of the zygoma, maxillary wall and crest, as well as the biomechanical behavior of the zygoma, including finite element analysis, is key to providing reliable treatment with zygomatic implants [48,49,50,51]. The ZAGA classification (Aparicio 2011), as well as the definition of three zones of atrophy (Bedrossian et al., 2008), is an example of such anatomical studies with direct implications for clinical planning (Aparicio, Polido and Zarrinkelk 2021). The definition of the Zygomatic Implant Critical Zone further supports its clinical impact [51]. Finally, anatomical bases for zygomatic implant placement are reported in the literature [52]. The use of surgical guides is regarded as a potential way to improve the accuracy and precision of implant placement. Real-time navigation techniques represent a research line to further reinforce the predictability and safety of this procedure. Surgical techniques are being thoroughly studied and enhanced. This includes the definition of the implant path and osteotomies.

The scope of these conclusions is limited by the low number of peer-reviewed publications with a clinical follow-up period greater than 10 years. This work represents a total of 3627 patients aggregated among 93 peer-reviewed publications. The results obtained are comparable to the 16 reviews screened. This study allowed us to reach several key points. The CSR over time of zygomatic implants to rehabilitate severe maxillary atrophy shows that this treatment option is safe and reliable. The complications reported by the authors represent key points where this treatment option can progress further to better help patients, and there is a need to use patient-centered success criteria, including more outcome measures than only implant survival [53].

Zygomatic implants represent an attractive treatment option for severe maxillary atrophy where edentulism and loss of bone are considered a handicap [54] and classified by WHO as a physical disability. Its rehabilitation aims to provide fixed teeth, function and aesthetics. Zygomatic implant therapy, with its high CSR over time, offers the additional patient-centered benefit of immediacy. Indeed, delivering a provisional set of teeth is possible in 8 to 24h. Zygomatic implants are known as an alternative to sinus lift procedures and bone grafting. Like general dental implants that are placed in alveolar bone, the zygomatic implants are supported in the basal facial structure that reaches zygomatic bone. Zygomatic implants can support a full arch permanently fixed bridge or a removable prosthesis such as a clip-on denture for the severely resorbed maxilla.

Immediate loading seems to be the choice for zygomatic implant as it allows early function and restoration. Therefore, after the implant stability evaluation post-surgery, the prosthesis is restored and for pathological occlusion in patients and the prosthetic design is customized for pronounced buccal inclination. However, in order to reduce the stress in the zygomatic implants and prosthetic screws, stiffer materials, such as cobalt chromium, zirconium and titanium, appear to be preferable options for stress pattern distribution in zygomatic implants [55].

The prevention and treatment of complications, especially rhinosinusitis, soft tissue and prosthetic complications, is an open challenge with several lines of ongoing research. Different approaches aim to reduce and prevent soft tissue complications with zygomatic implants, such as the scarf graft, the Bichat buccal fat pad or the use of flat ZAGA implants to reduce the stress on the soft tissue. Moreover, in some anatomical situations, the extra-maxillary technique allows maintaining the sinus membrane intact and thus avoids rhinosinusal complications. Finally, a prosthetic-driven implant positioning with an optimized biomechanical design of the prosthesis is expected to reduce prosthetic complications [56,57].

Almeida et al. [58] and Sartori et al. [59] focus on patient satisfaction after a zygomatic implant rehabilitation. Furthermore, the ORIS criteria include more dimensions to success than only implant survival. Indeed, patient satisfaction, rhinosinusal state and soft tissue conditions are parameters used to calculate the ORIS score at any given follow-up time.

A key limitation of this study is that the definition of the success rate of ZI varied among authors. Additionally, the use of different surgical techniques such as intra-sinus or extra-maxillary could also influence the CSR and complication rate overall. The patient satisfaction is mostly absent from the results reported. Losing a non-functional tooth may not affect an individual’s quality of life, but the implications of losing a tooth will have consequences for the rest of the dentition. With this being said, those who have severely resorbed bone in the maxilla are at a greater detriment, requiring advanced treatment(s) for their condition. Zygomatic implant (ZI) therapy is an elective treatment modality for the rehabilitation of partially and fully edentulous patients.

## 5. Conclusions

In conclusion, our findings show that zygomatic implants are a safe and reliable option for treating severe maxillary atrophy. The decrease in the cumulative success rate over time is minimal within the first 10 years of clinical follow-up, suggesting that zygomatic implants are a viable long-term option. The follow-up of reported complications, their prevention and treatment represent the next challenge, along with patient-centered success criteria, including implant survival, rhinosinus evaluation, soft tissue condition and patient satisfaction. Future clinical trials with consistency in consensual survival criteria development will be required for the evaluation of the long-term effectiveness of ZI implants and the soft and hard tissue response.

## Data Availability

Not applicable.

## References

[1] Brånemark P., Gröndahl K., Öhrnell L., Nilsson P., Petruson B., Svensson B., Engstrand P., Nannmark U. (2004). Zygoma Fixture in the Management of Advanced Atrophy of the Maxilla: Technique and Long-Term Results. Scand. J. Plast. Reconstr. Surg. Hand Surg..

[2] Chana H., Smith G., Bansal H., Zahra D.A. (2019). Chana, Harpal, Graham Smith, Harjot Bansal, and Daniel Zahra. A Retrospective Cohort Study of the Survival Rate of 88 Zygomatic Implants Placed Over an 18-Year Period. Int. J. Oral Maxillofac. Implant..

[3] Balan I., Di Girolamo M., Lauritano D., Carinci F. (2017). Treatment of severe atrophic maxilla with zygomatic implants: A case series. Oral Implantol..

[4] Bedrossian E. (2010). Rehabilitation of the edentulous maxilla with the zygoma concept: A 7-year prospective study. Int. J. Oral Maxillofac. Implant..

[5] Esposito M., Davó R., Marti-Pages C., Ferrer-Fuertes A., Barausse C., Pistilli R., Ippolito D.R., Felice P. (2018). Immediately Loaded Zygomatic Implants vs Conventional Dental Implants in Augmented Atrophic Maxillae: 4 Months Post-Loading Results from a Multicentre Randomised Controlled Trial. Eur. J. Oral Implantol..

[6] Aparicio C., Manresa C., Francisco K., Claros P., Alández J., González-Martín O., Albrektsson T. (2000). Zygomatic Implants: Indications, Techniques and Outcomes, and the Zygomatic Success Code. Periodontology.

[7] Bedrossian E., Stumpel L., Beckely M.L., Indresano T. (2002). The zygomatic implant: Preliminary data on treatment of severely resorbed maxillae. A clinical report. Int. J. Oral Maxillofac. Implant..

[8] Aparicio C., López-Piriz R., Albrektsson T. (2020). ORIS Criteria of Success for the Zygoma-Related Rehabilitation: The (Revisited) Zygoma Success Code. Int. J. Oral Maxillofac. Implant..

[9] Higgins J.P., Green S. (2011). Cochrane Handbook for Systematic Reviews of Interventions Version 5.

[10] Hirsch J.-M., Öhrnell L.-O., Henry P.J., Andreasson L., Brånemark P.-I., Chiapasco M., Gynther G., Finne K., Higuchi K.W., Isaksson S. (2004). A Clinical Evaluation of the Zygoma Fixture: One Year of Follow-up at 16 Clinics. J. Oral Maxillofac. Surg..

[11] Peñarrocha-Diago M., Bernabeu-Mira J.C., Fernández-Ruíz A., Aparicio C., Peñarrocha-Oltra D. (2020). Bone Regeneration and Soft Tissue Enhancement Around Zygomatic Implants: Retrospective Case Series. Materials.

[12] Davó R., Malevez C., Rojas J., Rodríguez J., Regolf J. (2008). Clinical Outcome of 42 Patients Treated with 81 Immediately Loaded Zygomatic Implants: A 12- to 42-Month Retrospective Study. Eur. J. Oral Implantol..

[13] Chow J., Wat P., Hui E., Lee P., Li W. (2010). A New Method to Eliminate the Risk of Maxillary Sinusitis with Zygomatic Implants. Int. J. Oral Maxillofac. Implant..

[14] Stiévenart M., Malevez C. (2010). Rehabilitation of Totally Atrophied Maxilla by Means of Four Zygomatic Implants and Fixed Prosthesis: A 6-s40-Month Follow-Up. Int. J. Oral Maxillofac. Surg..

[15] Migliorança R.M., Coppedê A., Rezende R.C.L.D., De Mayo T. (2011). Restoration of the Edentulous Maxilla Using Extrasinus Zygomatic Implants Combined with Anterior Conventional Implants: A Retrospective Study. Int. J. Oral Maxillofac. Implant..

[16] Maló P., Nobre M., Lopes A., Francischone C., Rigolizzo M. (2012). Three-year outcome of a retrospective cohort study on the rehabilitation of completely edentulous atrophic maxillae with immediately loaded extra-maxillary zygomatic implants. Eur. J. Oral Implantol..

[17] Peñarrocha M., Carrillo C., Boronat A., Martí E. (2007). Level of satisfaction in patients with maxillary full-arch fixed prostheses: Zygomatic versus conventional implants. Int. J. Oral Maxillofac. Implant..

[18] Esposito M., Barausse C., Balercia A., Pistilli R., Ippolito D.R., Felice P. (2017). Conventional drills vs piezoelectric surgery preparation for placement of four immediately loaded zygomatic oncology implants in edentulous maxillae: Results from 1-year split-mouth randomised controlled trial. Eur. J. Oral Implantol..

[19] Pellegrino G., Relics D., Tarsitano A., Basile F., Grande F., Marchetti C. (2018). Computer-Assisted Surgery in the Rehabilitation of the Upper Jaw with Zygomatic Implants-a Cohort Study Comparing Atrophic and Oncologic Patients. Preliminary Results at 4 Years Follow-Up. Clin. Oral Impl. Res..

[20] Wang C.I., Cho S.H., Ivey A., Reddy L.V., Sinada N. (2021). Combined bone and mucosa-supported 3D-printed guide for sinus slot preparation and prosthetically driven zygomatic implant placement. J. Prosthet. Dent..

[21] Hinze M., Vrielinck L., Thalmair T., Wachtel H., Bolz W. (2013). Zygomatic Implant Placement in Conjunction with Sinus Bone Grafting: The ‘Extended Sinus Elevation Technique. A Case-Cohort Study. Int. J. Oral Maxillofac. Implant..

[22] Borgonovo A., Grandi T., Vassallo S., Signorini L. (2021). Extrasinus Zygomatic Implants for the Immediate Rehabilitation of the Atrophic Maxilla: 1-Year Postloading Results From a Multicenter Prospective Cohort Study. J. Oral Maxillofac. Surg..

[23] Pellicer-Chover H., Cervera-Ballester J., Penarrocha-Oltra D., Bagan L., Penarrocha-Diago M. (2016). Influence of the Prosthetic Arm Length (Palatal Position) of Zygomatic Implants upon Patient Satisfaction. Med. Oral Patol. Oral Cir. Bucal.

[24] Araújo R., Sverzut A.T., Trivellato A., Sverzut C. (2017). Retrospective Analysis of 129 Consecutive Zygomatic Implants Used to Rehabilitate Severely Resorbed Maxillae in a Two-Stage Protocol. Int. J. Oral Maxillofac. Implant..

[25] Al-Thobity A., Wolfinger G., Balshi S., Flinton R., Balshi T. (2014). Zygomatic Implants as a Rehabilitation Approach for a Severely Deficient Maxilla. Int. J. Oral Maxillofac. Implant..

[26] Butura C.C., Galindo D.F. (2014). Combined Immediate Loading of Zygomatic and Mandibular Implants: A Preliminary 2-Year Report of 19 Patients. Int. J. Oral Maxillofac. Implant..

[27] Uchida Y., Goto M., Katsuki T., Akiyoshi T. (2001). Measurement of the Maxilla and Zygoma as an Aid in Installing Zygomatic Implants. J. Oral Maxillofac. Surg..

[28] de Moraes E.J. (2012). The buccal fat pad flap: An option to prevent and treat complications regarding complex zygomatic implant surgery. Preliminary report. Int. J. Oral Maxillofac. Implant..

[29] Atalay B., Doğanay Ö., Saraçoğlu B.K., Bultan Ö., Hafiz G. (2017). Clinical Evaluation of Zygomatic Implant-Supported Fixed and Removable Prosthesis. J. Craniofacial Surg..

[30] Gracher A.H.P., de Moura M.B., Peres P.D.S., Thomé G., Padovan L.E.M., Trojan L.C. (2021). Full Arch Rehabilitation in Patients with Atrophic Upper Jaws with Zygomatic Implants: A Systematic Review. Int. J. Implant Dent..

[31] Ramezanzade S., Yates J., Tuminelli F.J., Keyhan S.O., Yousefi P., Lopez-Lopez J. (2021). Zygomatic implants placed in atrophic maxilla: An overview of current systematic reviews and meta-analysis. Maxillofac. Plast. Reconstr. Surg..

[32] Muñoz D.G., Aldover C.O., Zubizarreta-Macho Á., Menéndez H.G., Castro J.L., Peñarrocha-Oltra D., Montiel-Company J., Montero S.H. (2021). Survival Rate and Prosthetic and Sinus Complications of Zygomatic Dental Implants for the Rehabilitation of the Atrophic Edentulous Maxilla: A Systematic Review and Meta-Analysis. Biology.

[33] Lan K., Wang F., Huang W., Davó R., Wu Y. (2021). Quad Zygomatic Implants: A Systematic Review and Meta-Analysis on Survival and Complications. Int. J. Oral Maxillofac. Implant..

[34] Lorusso F., Conte R., Inchingolo F., Festa F., Scarano A. (2021). Survival Rate of Zygomatic Implants for Fixed Oral Maxillary Rehabilitations: A Systematic Review and Meta-Analysis Comparing Outcomes between Zygomatic and Regular Implants. Dent. J..

[35] Sales P.H., Gomes M.V., Oliveira-Neto O.B., de Lima F.J., Leão J.C. (2020). Quality assessment of systematic reviews regarding the effectiveness of zygomatic implants: An overview of systematic reviews. Med. Oral Patol. Oral Cir. Bucal.

[36] Migliorança R.M., Irschlinger A.L., Peñarrocha-Diago M., Fabris R.R., Vicente J.A. (2019). History of zygomatic implants: A systematic review and meta-analysis. Dent. Oral Craniofac. Res..

[37] Alqutaibi A.Y., Aboalrejal A. (2017). Zygom Implants Are a Reliable Treatment Option for Patients With Atrophic Maxilla. J. Evid. Based Dent. Pract..

[38] Cid F., Soto B. (2016). Success Rate of Zygomatic Implants for Rehabilitation of Severely Atrophied Maxillae: A Review of the Literature Tasa de Éxito de Los Implantes Cigomáticos Para La Rehabilitación Del Maxilar Severamente Atrofiado: Una Revisión de La Literatura Success Rat. Int. J. Med. Surg. Sci..

[39] Chrcanovic B.R., Albrektsson T., Wennerberg A. (2016). Survival and Complications of Zygomatic Implants: An Updated Systematic Review. J. Oral Maxillofac. Surg..

[40] Wang F., Monje A., Lin G.H., Wu Y., Monje F., Wang H.L., Davó R. (2015). Reliability of four zygomatic implant-supported prostheses for the rehabilitation of the atrophic maxilla: A systematic review. Int. J. Oral Maxillofac. Implant..

[41] Goiato M.C., Pellizzer E.P., Moreno A., Gennari-Filho H., Dos Santos D.M., Santiago Jr J.F., Dos Santos E.G. (2014). Implants in the Zygomatic Bone for Maxillary Prosthetic Rehabilitation: A Systematic Review. Int. J. Oral Maxillofac. Surg..

[42] Prithviraj D.R., Vashisht R., Bhalla H.K. (2014). From Maxilla to Zygoma: A Review on Zygomatic Implants. J. Dent. Implant..

[43] Chrcanovic B.R., Pedrosa A.R., Custódio A.L.N. (2013). Zygomatic Implants: A Critical Review of the Surgical Techniques. Oral Maxillofac. Surg..

[44] Candel-Martí E., Carrillo-García C., Peñarrocha-Oltra D., Peñarrocha-Diago M. (2012). Rehabilitation of Atrophic Posterior Maxilla with Zygomatic Implants: Review. J. Oral Implant..

[45] Galán Gil S., Peñarrocha Diago M., Balaguer Martínez J., Marti Bowen E. (2007). Rehabilitation of Severely Resorbed Maxillae with Zygomatic Implants: An Update. Med. Oral Patol. Oral Cir. Bucal.

[46] Davó R., Pons O. (2015). 5-year outcome of cross-arch prostheses supported by four immediately loaded zygomatic implants: A prospective case series. Eur. J. Oral Implantol..

[47] Filho H.N., Amaral W.S., Curra C., Dos Santos P.L., Cardoso C.L. (2016). Zygomatic Implant: Late Complications in a Period of 12 Years of Experience. Rev. Clínica Periodoncia Implantol. Rehabil. Oral.

[48] Yalçın M., Can S., Akbaş M., Dergin G., Garip H., Aydil B., Varol A. (2020). Retrospective Analysis of Zygomatic Implants for Maxillary Prosthetic Rehabilitation. Int. J. Oral Maxillofac. Implant..

[49] Yates J., Brook I., Patel R., Wragg P., Atkins S., El-Awa A., Bakri I., Bolt R. (2014). Treatment of the Edentulous Atrophic Maxilla Using Zygomatic Implants: Evaluation of Survival Rates over 5–10 Years. Int. J. Oral Maxillofac. Surg..

[50] Zanna M., Mascitti M., Coccia E., Lo Muzio L., Santarelli A. (2018). Spider Zygoma: A new implant rehabilitation technique for atrophic maxilla. J. Biol. Regul. Homeost. Agent..

[51] Zhou W., Fan S., Wang F., Huang W., Jamjoom F.Z., Wu Y. (2019). A Novel Extraoral Registration Method for a Dynamic Navigation System Guiding Zygomatic Implant Placement in Patients with Maxillectomy Defects. J. Oral Maxillofac. Surg..

[52] Aparicio C., López-Píriz R., Peñarrocha M. (2021). Preoperative Evaluation and Treatment Planning. Zygomatic Implant Critical Zone (ZICZ) Location. Atlas Oral Maxillofac. Surg. Clin. N. Am..

[53] Nkenke E., Hahn M., Lell M., Wiltfang J., Schultze-Mosgau S., Stech B., Radespiel-Tröger M., Neukam F.W. (2003). Anatomic Site Evaluation of the Zygomatic Bone for Dental Implant Placement. Clin. Oral Impl. Res..

[54] Davis D.M., Fiske J., Scott B., Radford D.R. (2000). The Emotional Effects of Tooth Loss: A Preliminary Quantitative Study. Br. Dent. J..

[55] Heboyan A., Giudice R.L., Kalman L., Zafar M.S., Tribst J.P.M. (2022). Stress Distribution Pattern in Zygomatic Implants Supporting Different Superstructure Materials. Materials.

[56] Wang F., Bornstein M.M., Hung K., Fan S., Chen X., Huang W., Wu Y. (2018). Application of Real-Time Surgical Navigation for Zygomatic Implant Insertion in Patients with Severely Atrophic Maxilla. J. Oral Maxillofac. Surg..

[57] Nocini R., Panozzo G., Trotolo A., Sacchetto L. (2022). Maxillary Sinusitis as a Complication of Zygomatic Implants Placement: A Narrative Review. Appl. Sci..

[58] Almeida P.H., Salvoni A.D., França F.M. (2017). Evaluation of Satisfaction of Individuals Rehabilitated with Zygomatic Implants as Regards Anesthetic asnd Sedative Procedure: A Prospective Cohort Study. Ann. Med. Surg..

[59] Sartori E.M., Padovan L.E.M., Sartori I.A.D.M., Ribeiro P.D., Carvalho A.C.G.D.S., Goiato M.C. (2012). Evaluation of Satisfaction of Patients Rehabilitated with Zygomatic Fixtures. J. Oral Maxillofac. Surg..

